# Supplementary material for: Content-rich biological network constructed by mining PubMed abstracts
Source: BMC Bioinformatics. 2004 Oct 8;5:147. doi: 10.1186/1471-2105-5-147 (PMC528731; doi:10.1186/1471-2105-5-147)
Supplement: Additional File 2 — The original results of the above study (non-essential files are deleted to keep the file size under the limit set by BMC bioinformatics). [file 1471-2105-5-147-S2.bz2 › chilibotAdditionalFile2/dip30/441ID9169459E10393/html/CCR3_CCL11.html]

 


 **CCR3** and **CCL11** 
  
Found 222 abstracts in PubMed, retrieved 30.  
 

 What does Google say? 
 PDF only 
| .edu only 

---

**Interactive relationship** (e.g. stimulation, inhibition, etc)

**Neutral relationship**- Like eotaxin  [ **CCL11** ]  and eotaxin  [ **CCL11** ]  2, eotaxin  [ **CCL11** ]  3 mediates its activity through  **CCR3** .  Ref: 12689946 Blood< MedlineTA>Blood,
- An inhibitor of the eotaxin  [ **CCL11** ]   **CCR3**  binding interaction may have potential as an anti inflammatory drug for treatment of asthma, parasitic infections, and allergic disorders.  Ref: 12857386 J Biomol Screen, 2003
- N terminal domain of eotaxin  [ **CCL11** ]  3 is important for activation of CC chemokine receptor 3  [ **CCR3** ] .  Ref: 12538912 Protein Eng, 2002
- Competitive binding studies showed that the binding affinity of truncated mutant toward  **CCR3**  was almost the same as that of wild type eotaxin  [ **CCL11** ]  3 even though the N terminal truncation involved the first through to the ninth residues.  Ref: 12538912 Protein Eng, 2002
- BACKGROUND Eotaxin  [ **CCL11** ]  is a chemokine that binds with high affinity and specificity to the chemokine receptor  **CCR3**  and plays an important role in the pathogenesis of allergic disease.  Ref: 12789237 J Allergy Clin Immunol, 2003
- The Eotaxin  [ **CCL11** ]  gene family Eotaxin1, Eotaxin2 and Eotaxin3 recruits and activates  **CCR3**  bearing cells such as eosinophils, mast cells and Th2 lymphocytes that play a major role in allergic disorders.  Ref: 12761043 Hum Mol Genet< MedlineTA>Hum Mol Genet, 2003
- Eotaxin  [ **CCL11** ]  is a potent chemokine that acts via CC chemokine receptor 3  [ **CCR3** ]   **CCR3**  to induce chemotaxis, mainly on eosinophils.  Ref: 12413953 Biochem Biophys Res Commun, 2002
- The eotaxin  [ **CCL11** ]  group chemokines eotaxin  [ **CCL11** ] , eotaxin  [ **CCL11** ]  2, and eotaxin  [ **CCL11** ]  3 share only 35 41% sequence identity but are all agonists for the receptor  **CCR3** .  Ref: 12486712 Proteins, 2003
- Eotaxin  [ **CCL11** ] , an inducer of eosinophil migration and activation, exerts its activity by binding to  **CCR3** , the C C chemokine receptor 3  [ **CCR3** ] .  Ref: 12857386 J Biomol Screen, 2003
- Hence, eosinophil responses mediated by chemokines acting at  **CCR3**  may be regulated by two distinct mechanisms the antagonistic effects of CXCR3 ligands and the sequestration of  **CCL11**  by CXCR3 expressing cells.  Ref: 12884299 Eur J Immunol, 2003
- Eotaxin  [ **CCL11** ]  3 belongs to the CC chemokine family, and specifically recognizes CC chemokine receptor CCR 3  [ **CCR3** ]  that is expressed on eosinophils, basophils and helper T type 2 cells.  Ref: 12538912 Protein Eng, 2002
- This migrational response to eotaxin  [ **CCL11** ]  was abrogated by anti  **CCR3**  monoclonal antibody and primed by preincubation with IL 5.  Ref: 12871220 ImmunologyImmunology, 2002
- In contrast, CCL18 and I TAC interferon inducible T cell alpha chemoattractant were neutral antagonists, inhibiting eotaxin  [ **CCL11** ]  induced 35S GTPgammaS binding, with minimal effect on basal coupling of  **CCR3**  to G proteins.  Ref: 12450563 Eur J Pharmacol, 2002

**Non-interactive relationship** (e.g. studied together, co-existance, homology, etc.)

- Stably transfected Cynomolgus  **CCR3**  bound human eotaxin  [ **CCL11** ]   **CCL11**  with similar kinetics Kd 240 pM and was responsive to human  **CCR3**  ligands eotaxin  [ **CCL11** ]   **CCL11** , eotaxin  [ **CCL11** ]  2 CCL24, and MCP4 CCL13 in Ca 2 mobilization and chemotaxis assays, thus provides a useful alternative species model system for the analysis of modulators of eotaxin  [ **CCL11** ]   **CCR3**  induced signaling and activation.  Ref: 12421570 Cytokine, 2002
- Following treatment with the sulfation inhibitor chlorate or proteoglycanases, no inhibition of  **CCL11**  induced activity was observed using either eosinophils or a  **CCR3**  expressing transfectant cell line.  Ref: 12731055 Eur J Immunol, 2003
- Nevertheless, stimulation of hMCs with eotaxin  [ **CCL11** ]  1 2 h after Fc epsilon RI cross linkage concomitantly with maximal surface  **CCR3**  expression increased Fc epsilon RI dependent IL 13 generation by hMCs, compared with their replicates stimulated simultaneously with both agonists.  Ref: 12654630 Am J Respir Cell Mol Biol, 2003
- An eosinophil active chemokine, eotaxin  [ **CCL11** ] , and its receptor, C C chemokine receptor 3  [ **CCR3** ] , are particularly attractive as novel targets of immunological intervention for the disease.  Ref: 12842759 Cytokine< MedlineTA>Cytokine, 2003
- Results have shown that  **CCR3**  mediated eotaxin  [ **CCL11** ]  induced eosinophilic degranulation was concentration dependently reduced by specific inhibitors of ERK1 ERK2, p38 MAP kinase and PI 3 kinase.  Ref: 12784909 Immunopharmacol Immunotoxicol, 2003
- One exception was eotaxin  [ **CCL11** ] , a high affinity ligand for  **CCR3** , which bound to rhesus CCR2B receptor K i =1467 205pM.  Ref: 12826275 Biochem Pharmacol, 2003
- In contrast, the  **CCR3**  agonists  **CCL11** , CCL24 and CCL26 are involved in the recruitment of cells such as eosinophils and basophils during Th2 responses.  Ref: 12884299 Eur J Immunol, 2003
- The examination of  **CCR3**  CCR1 chimeric constructs revealed that  **CCL11**  and CXCL11 share overlapping binding sites contained within the  **CCR3**  extracellular loops, a region that was previously shown to be essential for effective receptor activation.  Ref: 12884299 Eur J Immunol, 2003
- The sources of IL 4, IL 5, eotaxin  [ **CCL11** ]  and RANTES and the expression of  **CCR3**  were examined by immunostaining of serial sections with antibodies to IL 4, IL 5, eotaxin  [ **CCL11** ] , RANTES and  **CCR3** , and antibodies to tryptase, ECP EG2 and CD3.  Ref: 12653173 Clin Exp Allergy, 2002
- Banyu I has a K d of 5.0 0.4 and 4.3 1.8 nM on human  **CCR3**  transfectants and eosinophils, and noncompetitively inhibits 125I eotaxin  [ **CCL11** ]  binding and eotaxin  [ **CCL11** ]  induced 35S guanosine 5 O 3 thiotriphosphate 35S GTPgammaS binding.  Ref: 12450563 Eur J Pharmacol, 2002
- The intragraft expression of interferon gamma IFN gamma, transforming growth factor beta1 TGF beta1, interleukin 4 IL 4, eotaxin  [ **CCL11** ]  and  **CCR3**  was assessed using competitive reverse transcription polymerase chain reaction RT PCR.  Ref: 12581766 J Heart Lung Transplant, 2003
- Despite there being no difference in expression of CC chemokine receptor 3  [ **CCR3** ] , the priming effect of RANTES and eotaxin  [ **CCL11** ]  was significantly enhanced in eosinophils from the patients.  Ref: 12797483 Eur Respir J, 2003
- Heparin inhibited intracellular calcium flux, respiratory burst and chemotactic responses of eosinophils to  **CCL11** , but not to the chemoattractant C5a, and inhibited binding of  **CCL11**  to  **CCR3** .  Ref: 12731055 Eur J Immunol, 2003
- METHODS We examined the number of tryptase cells, EG2 cells, CD3 cells, IL 4 cells, IL 5 cells, eotaxin  [ **CCL11** ]  cells, RANTES cells and  **CCR3**  cells in five specimens of KD versus normal tissues by immunohistochemistry.  Ref: 12653173 Clin Exp Allergy, 2002
- The truncated eotaxin  [ **CCL11** ]  3 proteins lacking the N terminal eight or nine residues inhibited the chemotactic activity of chemokines that recognize  **CCR3** .  Ref: 12538912 Protein Eng, 2002
- The eotaxin  [ **CCL11** ]  receptor  [ **CCR3** ]   **CCR3**  was expressed in both stromal and carcinoma cell populations of HL like UCNT, this pattern being similar to the one observed in HL.  Ref: 12792781 Int J Oncol, 2003
- Type 1 chemokines CXC chemokine ligand CXCL 9 and CXCL10 monokine induced by IFN gamma and IFN gamma inducible protein of 10 kDa CXCR3 ligands, and type 2 chemokines CC chemokine ligand CCL 11  [ **CCL11** ]  eotaxin  [ **CCL11** ]   **CCR3**  ligand, CCL17 thymus and activation regulated chemokine CCR4 ligand and CCL22 monocyte derived chemokine CCR4 ligand were readily detectable in the plasma of most participants.  Ref: 12407016 Int Immunol, 2002
- In particular, the eotaxin  [ **CCL11** ]  subfamily of chemokines and their receptor CC chemokine receptor 3  [ **CCR3** ]  have emerged as central regulators of the asthmatic response.  Ref: 12589338 J Allergy Clin Immunol, 2003
- Consistent with these results, infected cells from WT mice preferentially migrated in response to IP 10 CXCR3 ligand, while those from GKO mice migrated in response to eotaxin  [ **CCL11** ]   **CCL11**   **CCR3**  ligand.  Ref: 12875978 Am J Pathol, 2003
- The eosinophils demonstrated increased expression of major basic protein MBP, eotaxin  [ **CCL11** ] , and  **CCR3** , as well as increased necrotic death.  Ref: 12704143 Infect Immun, 2003
- To study mechanisms of progenitor cell mobilization in allergic reactions, we investigated fluctuations in the expression of the eotaxin  [ **CCL11** ]  receptor  [ **CCR3** ] , CC chemokine receptor 3  [ **CCR3** ]   **CCR3** , on CD34 cells from stable asthmatics following allergeni.e. antigen challenge.  Ref: 12871220 ImmunologyImmunology, 2003
- RESULTS Mast cells, activated eosinophils, T cells, IL 4 cells, IL 5 cells, eotaxin  [ **CCL11** ]  cells, RANTES cells and  **CCR3**  cells were all increased in the lesions of KD as compared with those in normal tissue.  Ref: 12653173 Clin Exp Allergy, 2002
- The chemokine eotaxin  [ **CCL11** ]  is involved in the recruitment of eosinophils and T helper 2 lymphocytes in human allergic diseases, and drugs that block its activity, including eotaxin  [ **CCL11** ]  receptor  [ **CCR3** ]   **CCR3**  antagonists, are being developed.  Ref: 12503787 Vet Rec, 2002
- **CCR3**  spontaneously couples to G proteins in  **CCR3**  transfectants, demonstrated by changes in basal and eotaxin  [ **CCL11** ]  induced 35S GTPgammaS binding under reduced NaCl and GDP concentrations.  Ref: 12450563 Eur J Pharmacol, 2002
- A radioligand binding assay was developed using HEK cells transfected with  **CCR3** , with 125 I eotaxin  [ **CCL11** ]  as the ligand.  Ref: 12857386 J Biomol Screen, 2003
